# Relationship of knob morphometric analysis with production performance and meat quality in Yangzhou goose (*Anser cygnoides*)

**DOI:** 10.3389/fphys.2023.1291202

**Published:** 2023-11-08

**Authors:** Yang Zhang, Xinlei Xu, Wangyang Ji, Shangzong Qi, Qiang Bao, Zhi Cao, Wei Liu, Yong Zhang, Yu Zhang, Qi Xu, Guohong Chen

**Affiliations:** ^1^ Key Laboratory for Evaluation and Utilization of Poultry Genetic Resources of Ministry of Agriculture and Rural Affairs, Yangzhou University, Yangzhou, China; ^2^ Yangzhou Tiangge Goose Industry Development Company Limited, Yangzhou, China

**Keywords:** knob, meat quality, packaging traits, production efficiency, Yangzhou geese

## Abstract

The development of the knob in Chinese geese (*Anser cygnodies*) is an outcome of extensive and prolonged selection and breeding. The knob serves not only as a visual indicator of sexual maturity in geese but also holds significance as a crucial packaging trait that attracts attention of consumers attentions, who tend to distinctly prefer geese with larger knobs. Consequently, investigating the formation of the knob holds practical value, as it will help achieving external traits aligned with consumers’ preferences. To understand the relationship between knob size, production efficiency, and meat quality in Yangzhou geese, we examined histological and anatomical characteristics in 500- and 120-day-old geese with large and small knobs. Notably, knob size had a pronounced impact on key anatomical and structural parameters, such as chest depth, leg muscle water-binding capacity, and insoluble collagen composition in Yangzhou geese (*p* < 0.05). In addition, we measured testosterone and estrogen levels in male and female geese, respectively, as well as growth hormone, and found that birds of both sexes with a large knob had higher sex and growth hormone levels in the body. This study established a fundamental theoretical basis for advancing the enhancement of goose knob traits.

## Introduction

China is the largest goose-producer in the world, with annual production reaching 4.29 million tons in 2021 (FAO-STAT., 2023). Although goose meat is mentioned as the top of the World Health Organization’s “Healthy Food List,” increasing goose consumption remains a persistent challenge (Akhtar et al., 2021).

In geese, the knob located posterior to the beak serves as an indicator of sexual maturity (Hudson et al., 2017) but it is also a vital packaging trait that captures consumers’ attention (Gumułka and Rozenboim, 2013; Ji et al., 2021). The presence and appearance of the knob, therefore, is a distinctive factor in goose sales that enables consumers to readily discern the gender, age, and other attributes of the birds.

The various resources are mainly distributed in Europe and Asia, and can be roughly classified into European geese and Chinese geese (Moniem et al., 2020). Compared with European geese, Chinese geese have a slender neck and grow into adults with a fleshy bulge at the top of the bill, known as a knob, which is a distinguishing feature (Zhang et al., 2023). On the whole, knobs tend to be larger in males than in females, and they are consistently larger in adults than in juveniles (Lee, 2021). Furthermore, at the same age, a larger knob indicates greater closeness to maturity and better meat quality (Weng et al., 2021; Razmaitė et al., 2022). Hence, investigating the relationship between knob formation and production performance is of practical significance for harnessing and maximizing the potential of this trait, ultimately contributing to the growth and promotion of goose meat consumption (Zhang et al., 2014; Liu et al., 2019; Ni et al., 2022). The dimensions of the knob are predominantly determined by the breed, age, and sex of geese, as well as by various other factors (Mawdesley-Thomas et al., 1967; Wright et al., 2012). Nonetheless, the knob size significantly influences production performance and meat quality metrics, an aspect that remains unexplored in the existing literature.

In this study, we characterized 120-day-old Yangzhou geese and performed an in-depth analysis of their histological and anatomical properties depending on the knob size. The primary objective was to elucidate the relationship between the knob size and the production performance and meat quality of Yangzhou geese. The ultimate aim was to foster the refinement, application, and preservation of the desired goose knob traits. These efforts have successfully established a robust theoretical foundation for driving future advancements in goose breeding (Yang et al., 2005).

## Materials and methods

### Ethics statement

All animal experiments were approved by the Animal Care and Use Committee of the Yangzhou University (approval number: 132-2020). The study was conducted in accordance with the local legislation and institutional requirements.

### Animals and sample collection

The geese were sourced from the Yangzhou Tiange Goose Industry Development Co., Ltd. (Yangzhou, Jiangsu, China). This experiment was performed using standard group breeding, and we then selected our target group from the standard group. The space of each goose house was maintained at 40 × 20 m, and the number of geese in each pen was maintained at approximately 500. The goose houses were semi-enclosed and received natural light during the day and artificial light at night from 19:00 to 21:00. In addition, each goose house was equipped with a pool designed to meet the water-playing habits of the geese and ensure animal welfare to a certain extent. A staff member regularly changed the water in the pool to ensure the hygiene and cleanliness of the water and reduce the occurrence of diseases. Usually, a combination of concentrated roughage and green fodder is used to supply the goose house, allowing the geese to eat freely. Concentrated roughage included standardized concentrate feed, corn, rice husks, and various mineral and vitamin preparations, while green feed was grown by the goose factory itself, harvested on time, and then fed in the goose houses for the geese to eat freely.

Once they met the market standard at 120 days, 15 geese with large knobs and 15 with small knobs, comprising a total of 30 individuals (*n* = 15), were selected for Experiment One. This subset of geese was utilized for the investigation of the correlation between knob size variations and performance, bone structure, histological characteristics, and meat quality. In order to determine knob and beak size, we established a set of methods for the determination of knob and beak sizes of mute swans in China (Horrocks et al., 2009), which has been published (Ji et al., 2021). The length, height, and width of the knob were measured in millimeters. The knob length (Kl) was measured between the most anterior part of the knob and the phenotypic boundary of the knob. The knob width (Kw) was measured at the widest part of the knob. The knob height (Kh) was measured between the fronto-nasal junction and the most dorsal part of the knob. Knob size was assessed using the product of knob length, width, and height. The criteria for classifying knob size for the 120-day-old geese were defined as large knobs having a volume (V) > 250,000 mm^3^ and small knobs having V < 15,000 mm^3^. For the 500-day-old geese, large knobs were defined as V > 40,000 mm^3^ and small knobs as V < 25,000 mm^3^. Before slaughter, the geese underwent a 12 h fasting period, after which they were euthanized via neck bleeding. After slaughtering, the head of each goose was removed. Subsequently, follicle-free skin derivatives from the knob (or forehead) of each goose’s head was extracted and fixed in 4% paraformaldehyde. The remaining portion was sealed in plastic, the appropriate label was applied, and it was stored in a −20°C refrigerator. Afterwards, the breast and leg muscles were meticulously dissected using a scalpel. The entire leg and chest muscles were isolated separately. A standard sample was chosen for hematoxylin and eosin (HE) tissue sectioning, and the remaining meat samples were sealed in bags and stored undisturbed at 4°C for 24 h to assess meat quality. The 24-h resting period is used to neutralize the meat’s acidity. Following the completion of correlation testing, individual samples undergo an immediate, secure handling process (Baéza et al., 2022). Experiment Two: selected 50 large-knob female geese and 50 small-knob female geese, and paired each group with 10 male geese with typical knob size. They were raised under identical feeding conditions until they reached 300 days of age, at which point we initiated measurements. These relevant performance evaluations continued until they reached 500 days of age. The primary objective of this study was to investigate the relationship between knob size and egg-production performance, as well as to assess the impact of knob size on hormone levels within the body.

### Blood collection

Before commencing the sampling work, we first conducted blood collection and weighing. The geese were restrained in the supine position, their wings were spread, and the inferior wing vein was exposed, wiped, and disinfected with alcohol, whereupon 1–2 mL of blood was collected into an anticoagulant vacuum blood collection tube and allowed to stand on ice for 30 min. Then, the tubes were centrifuged at 3,000 rpm for 15 min, and the upper layer of yellow transparent liquid was collected and stored at −20°C for use (Mewis et al., 2014).

### Measurements of body weight and size in geese with large and small knobs

The geese were weighed before slaughter. Body size measurements, specifically body oblique length, chest width, chest depth, sternal length, back width, tibial length, tibial circumference, semi-diving length, and neck length, were performed according to NY/T823-2004.

### Determination of knob histological index

Knob morphological traits were determined using HE staining (Ji et al., 2021). The skin samples were fixed in 4% paraformaldehyde for 24 h. The samples were placed in an embedding cassette and rinsed with running water for 30 min to remove the fixative from the tissue, and the samples were dehydrated using a graded ethanol series. The paraffin blocks were cut (Leica Biosystems, Wetzlar, Germany) along the horizontal axis into 3 μm thick sections and stained with HE according to standard protocols. The samples were scanned using a NanoZoomer scanner (Hamamatsu, Sydney, Australia). The fiber diameter and cross-sectional area were calculated using an image analysis system (Image-Pro Plus, Media Cybernetics, Rockville, MD, United States) (Zhang et al., 2023).

### Determination of meat quality in geese with large and small knobs

#### Tenderness determination

Tenderness was assessed using shear-force measurements. Based on previously selected fresh samples, portions of the chest and leg muscles were selected and sliced into elongated meat strips measuring 1.0 cm in width and 0.5 cm in thickness, aligned with the muscle fiber direction, and cleaned of any fascia or fat. Using a C-LM3 muscle tenderness instrument, meat samples were sliced along the vertical muscle fibers, and each sample was subjected to three cuts for shear-force measurement. The resulting shear force values were averaged to determine the tenderness (Geldenhuys, et al., 2014).

#### Determination of expressible water

The expressible water was determined as follows, A total of 1 g of tissue sample (m1) was dissected from the breast and leg muscls, using surgical scissors to trim them. The meat samples were sandwiched between 16 layers of filter paper, with an additional hard plastic backing plate placed on the outermost filter paper layer. Using a steel ring, a pressure of 35 kg was applied to the dilatometer platform for 5 min. After the application of pressure, the weight of the compressed meat samples (m2) was promptly recorded. The expressible water was calculated using the following formula: expressible water (%) = (m1–m2) × 100%/m1.

#### Meat color determination

Meat color was assessed at 24 h post-slaughter using a CR-400 colorimeter to measure the surface attributes of chest and leg muscles. In particular, values for redness (a*), yellowness (b*), and brightness (L*) were recorded, L* value was unaffected by bloom time; hue angle stabilized after 5 min, a* and b* values after 10 min and chroma after 20 min (Brewer et al., 2001; Weng et al., 2021).

#### pH determination

The procedure of pH measurement involved creating three incisions at distinct points along the cross-sectional surface of the chest and leg muscles of the carcass using a scalpel. Then, the electrode of a pen-type pH meter (specifically, a waterproof pH Spear test pen) was inserted to a penetration depth of 1 cm into the muscle tissue. The pH values of both breast and leg muscles were read three times immediately after slaughter, and the average value was calculated.

#### Determination of protein, fat, insoluble collagen, and water contents

The composition of thoracic and leg muscle samples was measured three times using a *Food Scan* rapid analyzer as described previously (Soren and Biswas, 2020). It primarily employs the near-infrared measurement principle, utilizing near-infrared spectroscopy technology to accurately quantify the content of the measured substance in meat products. This process avoids sample destruction or chemical treatment by analyzing signals within the spectral data associated with the absorption characteristics of the measured substance. Consequently, this method proves highly valuable for enhancing meat quality control and quality testing.

### Egg production and fertilization rate of geese with large and small knobs

This article selected 50 female geese with varying knob sizes and paired them with 10 male geese of typical knob size. Our objective was to allow them to naturally mate, facilitating the production of fertilized eggs for our later experiments and observations. They were raised under identical feeding conditions until they reached 300 days of age, at which point we initiated measurements. These relevant performance evaluations continued until they reached 500 days of age.

### Determination of hormone levels in geese with large and small knobs

To estimate hormone levels, blood was centrifuged, and serum was collected. The main hormones detected were the following: female geese, estrogen and growth hormone (GH) and male geese, androgen and GH levels by using commercially available enzymelinked immunosorbent assay (ELISA) kits (Wuhan EIAab Science Co., Ltd., Wuhan, Hubei, China). Operations were carried out according to the manufacturers’ protocols. No cross-reaction with other structural analogues, and all the intra-assay and inter-assay coefficients of variation (CV) for each hormonal assay were less than 10% and 15%. Finally, the absorbance of 450 nm was read for each well by a microplate reader, Model 680 (BioRad, Hercules, CA, United States).

### Statistics and analysis

The test data were initially processed using Excel, and Student’s t-test was used to analyze the significance of differences between knob size traits using SPSS 25.0. The results were expressed as the mean ± standard deviation.

## Results

### Knob skin thickness and bone protrusion parameters in geese with large and small knobs

**FIGURE 1:**
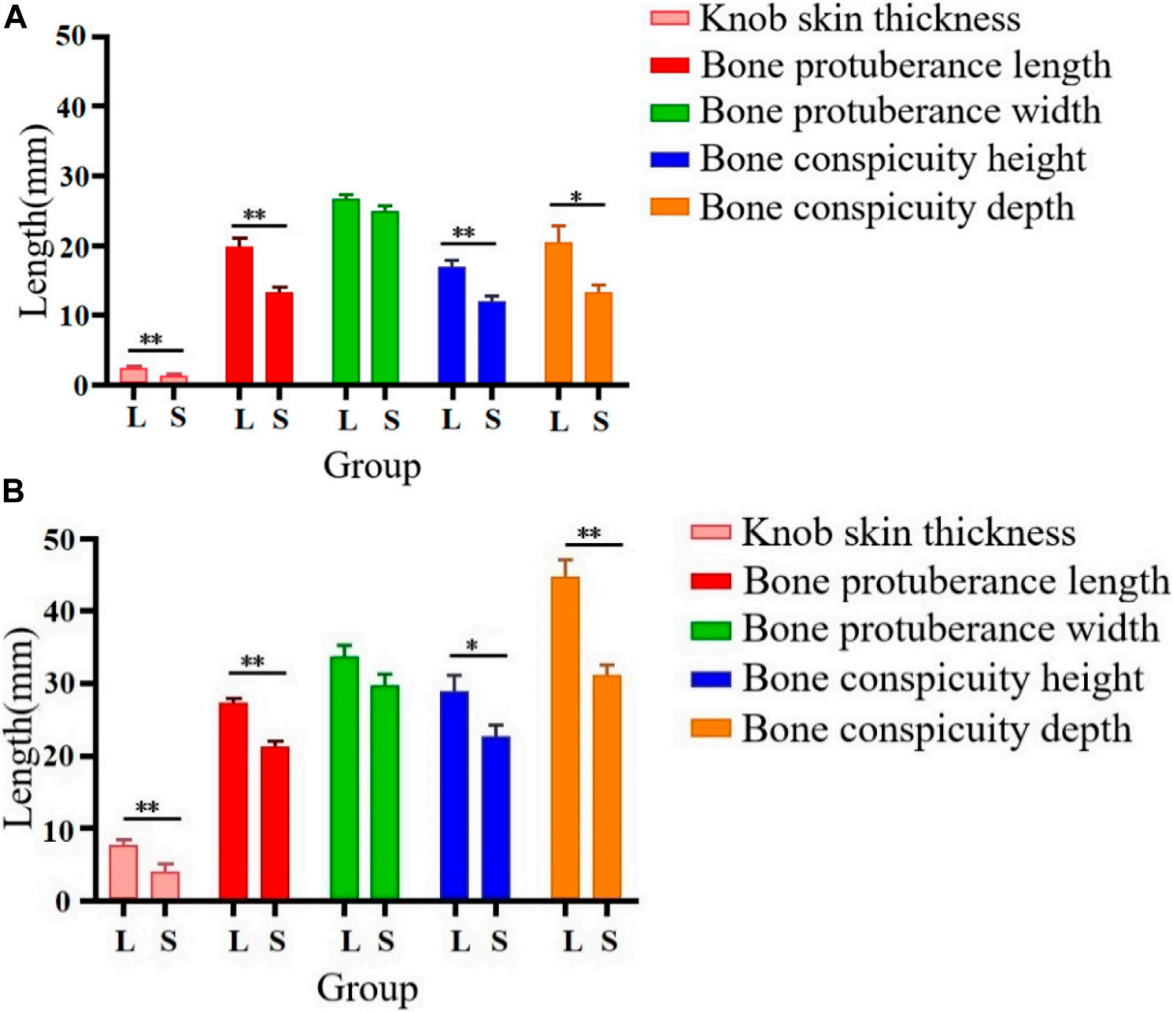
Knob skin thickness and bone characteristics in Yangzhou geese with large and small knobs. Heads of geese were collected to measure the knob skin thickness as well as the length, width, height, and depth of the bone protrusion. **(A)** 120 days of age. **(B)** 500 days of age. D, large-knob group; X, small-knob group.

As illustrated in Figure 1, we examined 120- and 500-day-old Yangzhou geese with large and small knobs. The criteria for classifying knob sizes were as follows: for the 120-day-old geese, large knobs were defined as volume (V) > 250,000 mm^3^ and small knobs as V < 15,000 mm^3^. For the 500-day-old geese, large knobs were defined as V > 40,000 mm^3^ and small knobs as V < 25,000 mm^3^. A comparative analysis was conducted on parameters such as the thickness of the overlying knob skin and the length, width, height, and depth of the bone protrusion. Bone protrusion length, height, and depth were significantly different in the groups of geese with large and small knobs (*p* < 0.05 in each case), whereas no significant knob-group effect was observed on the width of the bone protrusion (*p* > 0.05). Moreover, a noteworthy correlation was observed between knob size and bone protrusion size (*p* < 0.05), emphasizing that the dimensions of the bone protrusion hold a decisive role in determining knob size (Table 1).

**TABLE 1:** Correlation coefficients between knob and bone protuberance dimensions.

Parameter	Age	Knob length	Knob width	Knob height	Tumor skin thickness	Knob volume
Bone protuberance length	D120	0.964**	0.579	0.708*	0.835**	0.882**
	D500	0.844**	0.593	0.818**	0.897**	0.912**
Bone protuberance width	D120	0.438	0.35	0.537	0.179	0.543
	D500	0.466	0.955**	0.215	0.492	0.666*
Bone conspicuity height	D120	0.527	0.315	0.767**	0.739*	0.688*
	D500	0.300	0.145	0.833**	0.728*	0.456
Bone conspicuity depth	D120	0.548	0.503	0.794**	0.433	0.745*
	D500	0.706*	0.702*	0.765**	0.867**	0.861**
Bone conspicuity volume	D120	0.826**	0.519	0.809**	0.836**	0.873**
	D500	0.678*	0.623	0.852**	0.913**	0.842**

*p < 0.05, and **p < 0.01.

### Histological differences between large and small knobs

**FIGURE 2:**
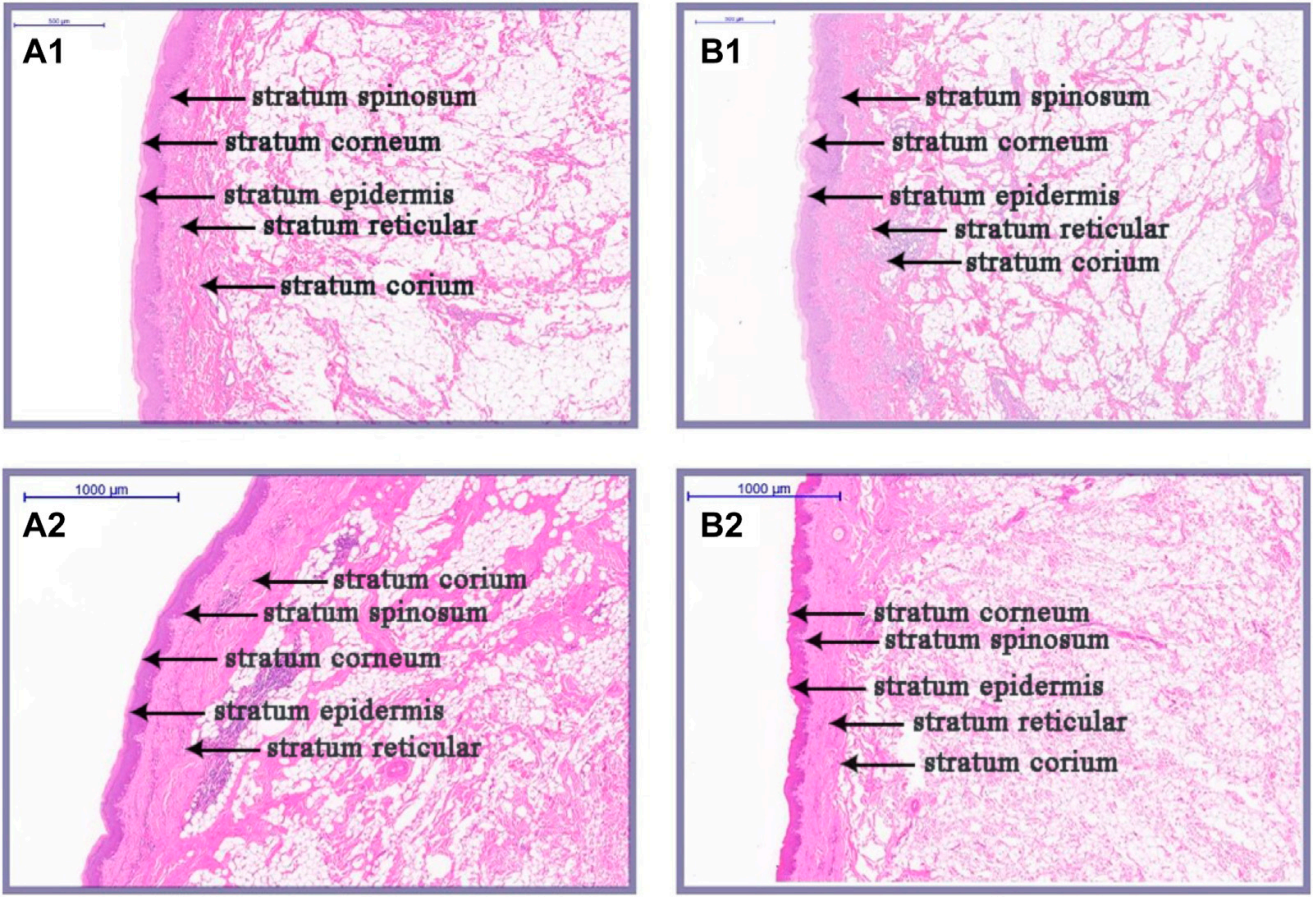
Histological images of large and small knobs in 120- and 500-day-old Yangzhou geese. Skin tissues of large and small knobs were collected from 120- and 500-day-old geese, fixed, embedded in paraffin and sectioned for staining with hematoxylin/eosin (magnification ×5). **(A1)** Large-knob skin tissue, 120 days. **(B)** Small-knob skin tissue, 120 days. **(A2)** Large-knob skin tissue, 500 days. **(B2)** Small-knob skin tissue, 500 days.

**FIGURE 3:**
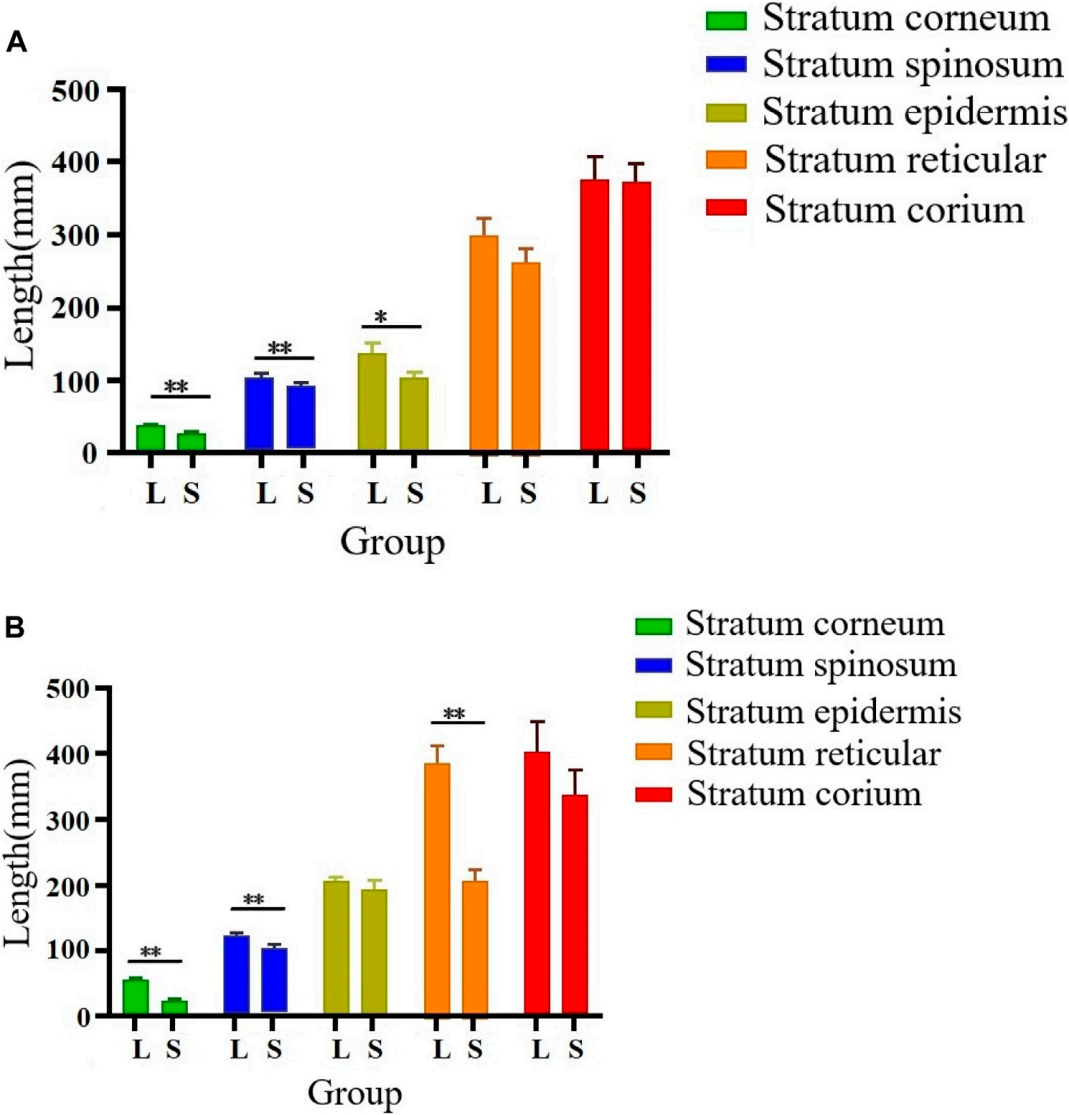
Morphometric comparison of large and small knobs in 120- and 500-day-old Yangzhou geese. Each layer in the knob skin tissue was carefully observed, and the length of each layer was measured. X-axis represents different layers; Y-axis represents layer length (in mm). **(A)** Histological differences in 120-day-old geese. **(B)** Histological differences in 500-day-old geese. L, large-knob group; S, small-knob group. Statistical significance of differences is illustrated as follows: **p < 0.01, and *p < 0.05.

As illustrated in Figures 2, 3, there were some histological differences between the large and small knobs in Yangzhou geese. Specifically, among the 120-day-old geese, histological differences were predominantly observed within the epidermal layer. In the case of large knobs, both the horny and spinous cell layers were remarkably expanded compared to the dimensions of these layers in small knobs (Figures 2A1, B1, 3A; *p* < 0.01). Moreover, the epidermal layer in geese with large knobs was significantly more extensive than that in geese with small knobs (Figures 2A1, B1, 3A; *p* < 0.05). In 500-day-old geese, similar histological changes were detected. Large knobs were characterized by significantly augmented dimensions in the horny, acanthocyte, and reticular layers compared to those in small knobs (Figures 2A2, B2, 3B; *p* < 0.01).

### Anatomical characteristics and production performance in geese with large and small knobs

As indicated in Table 2, a comprehensive statistical analysis was conducted on the body weight, body size, and post-slaughter measurements of 120-day-old Yangzhou geese with large and small knobs. Notably, among the examined parameters, the chest depth of geese with small knobs was significantly larger than that of geese with large knobs (*p* < 0.05), whereas no significant differences were observed in other body dimensions (Table 3).

**TABLE 2:** Production performance and carcass quality.

Parameter	Small knob	Large knob
Body weight, kg	4.42 ± 0.10	4.56 ± 0.11
Pec heavy, g	374.52 ± 17.03	407.35 ± 16.19
Leg muscle weight, g	350.14 ± 21.65	410.15 ± 22.60
Body slope length, cm	29.20 ± 1.31	29.23 ± 1.06
Chest width, cm	16.50 ± 0.22	16.00 ± 1.02
Chest depth, cm	14.20 ± 0.26^a^	12.45 ± 0.74^b^

**TABLE 3:** Effects of different knob sizes on goose performance.

Character	Small knob	Large knob
Sternum length, cm	17.62 ± 0.43	18.03 ± 0.94
Back width, cm	6.40 ± 0.43	6.70 ± 0.99
Tibial length, cm	9.36 ± 0.19	9.25 ± 0.32
Tibial circumference, cm	5.10 ± 0.19	5.20 ± 0.12
Half-submerged length, cm	81.20 ± 1.90	81.75 ± 1.30
Neck length, cm	29.40 ± 0.91	29.88 ± 1.42

Values in the same column marked by different uppercase letters are significantly different (*p* < 0.05). *n* = 30.

### Meat quality indices of geese with small or large knobs

Table 4, 5 presents the results of the Meat quality indices of the geese with small or large knobs. Notably, we found that the leg muscles of geese with large knobs had a significantly higher hydraulic value compared to that of the leg muscles of geese with small knobs (*p* < 0.05). Similarly, the insoluble insoluble collagen content of the leg muscles in geese with large knobs was significantly higher than that in the leg muscles of geese with small knobs (*p* < 0.05). However, no other parameters were significantly different in geese with large and small knobs.

**TABLE 4:** Meat quality indices of the geese with small or large knobs.

Parameter		Flesh			PH	Shear force	Expressible water
		L*	a*	b*			
Small knob	pectoralis	39.35 ± 2.42	15.49 ± 2.14	5.50 ± 1.55	6.26 ± 0.05	6.26 ± 8.26	0.31 ± 0.06^a^
	leg muscles	42.23 ± 5.24	17.25 ± 2.16	8.77 ± 1.21	6.13 ± 0.07	46.50 ± 9.92	0.21 ± 0.02^b^
Large knob	pectoralis	39.14 ± 0.81	13.74 ± 0.34	4.67 ± 0.6	6.12 ± 0.15	6.12 ± 10.61	0.33 ± 0.02^a^
	leg muscles	43.21 ± 5.62	14.14 ± 3.58	5.04 ± 1.45	6.13 ± 0.16	38.15 ± 4.53	0.32 ± 0.01^a^

**TABLE 5:** Meat quality indices of the geese with small or large knobs.

Character	Small knob		Large knob	
	Pectoralis	Leg muscles	Pectoralis	Leg muscles
Moisture	69.46 ± 0.26	71.41 ± 0.50	69.65 ± 0.47	71.20 ± 0.59
Protein	22.15 ± 0.22	21.32 ± 0.16	22.58 ± 0.38	21.51 ± 0.26
Fat	22.15 ± 0.34	4.15 ± 0.29	22.58 ± 0.52	3.36 ± 0.72
Insoluble collagen	0.22 ± 0.05	0.32 ± 0.05^b^	0.41 ± 0.07	0.50 ± 0.04^a^

a*, redness; b*, yellowness; L*, brightness. There is no significant difference between values with the same letters in the same column (*p* > 0.05), and the difference between values with different letters in the same column is significant (*p* < 0.05).

### Egg production performance in geese with large and small knobs

**FIGURE 4:**
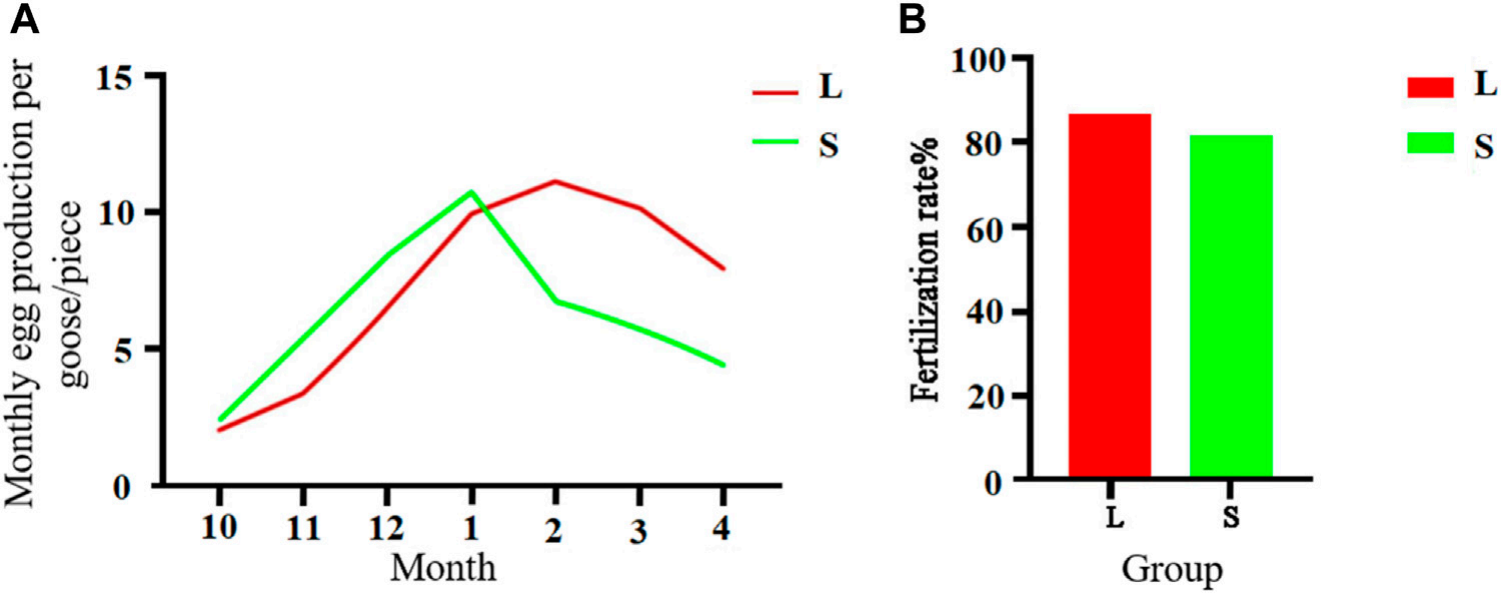
Monthly egg production **(A)** and egg fertilization rates **(B)** of female geese with different knob sizes. Monthly egg production in October–April. Overall egg fertilization rate. L, large-knob group; S, small-knob group.

A comparison between 300-day-old geese with large and small knobs showed interesting egg production patterns (Figure 4A). From October to January, the large-knob geese showed lower monthly egg production, whereas from February to April, egg production was higher than that in the small-knob geese. Notably, the small-knob geese began egg-laying earlier and achieved peak egg production in January. In contrast, the large-knob geese began laying eggs later and reached peak egg production in February. As illustrated in Figure 4B, of the 650 eggs collected from large-knob geese, 564 were fertilized, indicating a fertilization rate of 86.77%. In the small-knob group, 528 of 650 eggs were fertilized, with a fertilization rate of 81.23%. Although hens with large knobs showed nominally higher fertilization rates than those in small-knob hens, the observed difference did not reach the set level of statistical significance (*p >* 0.05).

### *In vivo* hormone concentrations in geese with large and small knobs

**FIGURE 5:**
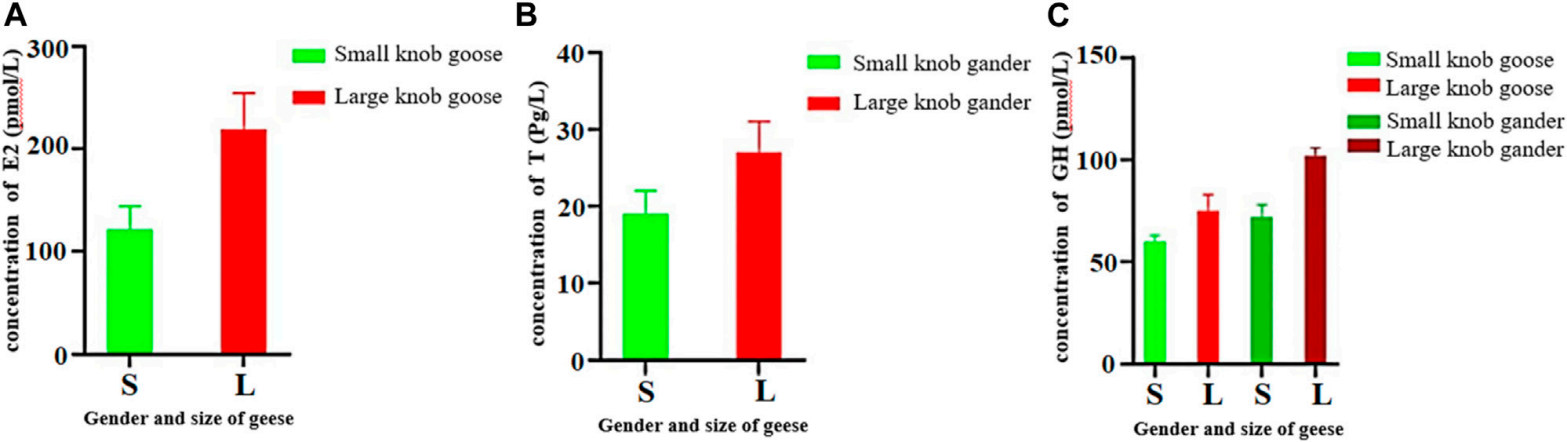
Hormone concentrations in 500-day-old geese with large and small knobs. Yangzhou geese at the age of 500 days were divided into groups of large-knob males, small-knob males, large-knob females, and small-knob females. Serum was collected, and estrogen (E2) and growth hormone (GH) levels in female geese and testosterone (T) and GH levels in male geese were measured. **(A)** Serum E2 levels in 300-day-old female geese. **(B)** Serum T levels in 300-day old male geese. **(C)** Serum GH levels in 300-day-old geese plotted separately for males and females. Statistical significance of differences is illustrated as follows: **p < 0.01, and *p < 0.05.

Female geese with small knobs had significantly lower estrogen concentrations than their larger knob counterparts (Figure 5A; *p* < 0.05). Similarly, testosterone concentrations were significantly lower in male geese with small knobs than in those with large knobs (Figure 5B; *p* < 0.05). Growth hormone levels were relatively consistent between male and female geese, with females having lower levels than males. Notably, geese with small knobs had significantly lower growth hormone levels compared to those in geese with large knobs (Figure 5C; *p* < 0.05).

## Discussion

In this study, we aimed to uncover and analyze the anatomical and histological differences between geese with large and small knobs at the ages of 120 and 500 days. In particular, we explored differences in meat quality parameters, body dimensions, egg-laying performance, hormone levels, and knob dimensions in birds with large and small knobs (Djermanovic et al., 2021). We observed that knob thickness as well as bone protrusion length, height, and depth were significantly different between large and small knobs. The histological discrepancies observed in Yangzhou geese with varying knob sizes underscore noteworthy distinctions. Irrespective of age (either 120 or 500 days), both the horny and spinous cell layers of large knobs were significantly thicker than those of small knobs (*p* < 0.05). Remarkably, in the case of 500-day-old geese, the reticular layer thickness in large knobs was significantly thicker than that in small knobs (*p* < 0.05). This layer predominantly consists of robust insoluble collagen and elastic fiber bundles that could conceivably influence the resilience of the skin overlying the knob. The insoluble collagen fibers within the reticular layer are interconnected with the subcutaneous tissues, suggesting that the thickness of this layer may also affect the thickness of the subcutaneous tissue.

Goose meat has exceptional nutritional value, with a rich protein content and minimal fat level. Consumers tend to be highly sensitive to meat quality while being less influenced by price fluctuations. Therefore, carcass quality is a pivotal criterion in goose breeding. In this study, we showed that at 120 days of age, male geese with larger knobs had significantly smaller breast depths than their small knob counterparts (*p* < 0.05). In this experiment, this paper used near-infrared FOODSCAN equipment for measurement. Near-infrared spectroscopy technology can quantitatively determine the insoluble collagen content in meat products without destroying the sample or chemically processing it by analyzing signals in the spectral data that are related to the absorption properties of insoluble collagen. Insoluble collagen plays an essential role in the connective tissue, serving as an irreplaceable factor in supporting the functions of both muscles and bones (Weston et al., 2002). The moisture content within the muscle tissue directly affects its succulence and palatability. Rearing geese under full grazing conditions enhances the moisture content of goose meat (Liu et al., 2011; Song et al., 2017). The findings of this study revealed that the leg muscles of geese with large knobs had higher moisture content, suggesting that selecting geese with larger knobs could indirectly foster the development of geese with heightened meat moisture content (Weng et al., 2021).

## Conclusion

This study delved into the histological and anatomical characteristics of Yangzhou geese at two different ages, 500 days and 120 days, encompassing both those with large and small knobs. Our findings unveiled a crucial connection between bone protrusion size and knob size, indicating that the former can significantly influence the latter. Furthermore, Geese possessing larger knobs exhibited traits associated with precocial puberty.

## Data Availability

The original contributions presented in the study are included in the article/supplementary material, further inquiries can be directed to the corresponding author.
